# RNA helicase DEAD-box-5 is involved in R-loop dynamics of preimplantation embryos

**DOI:** 10.5713/ab.23.0401

**Published:** 2024-02-22

**Authors:** Hyeonji Lee, Dong Wook Han, Seonho Yoo, Ohbeom Kwon, Hyeonwoo La, Chanhyeok Park, Heeji Lee, Kiye Kang, Sang Jun Uhm, Hyuk Song, Jeong Tae Do, Youngsok Choi, Kwonho Hong

**Affiliations:** 1 Department of Stem Cell and Regenerative Biotechnology, Institute of Advanced Regenerative Science, Konkuk University, Seoul 05029, Korea; 2 Guangdong Provincial Key Laboratory of Large Animal Models for Biomedicine, Wuyi University, Jiangmen 529020, China; 3 Department of Animal Science, Sangji University, Wonju 26339, Korea

**Keywords:** DEAD-box-5 (DDX5), Gene Transcription, R-loop, Zygote

## Abstract

**Objective:**

R-loops are DNA:RNA triplex hybrids, and their metabolism is tightly regulated by transcriptional regulation, DNA damage response, and chromatin structure dynamics. R-loop homeostasis is dynamically regulated and closely associated with gene transcription in mouse zygotes. However, the factors responsible for regulating these dynamic changes in the R-loops of fertilized mouse eggs have not yet been investigated. This study examined the functions of candidate factors that interact with R-loops during zygotic gene activation.

**Methods:**

In this study, we used publicly available next-generation sequencing datasets, including low-input ribosome profiling analysis and polymerase II chromatin immunoprecipitation-sequencing (ChIP-seq), to identify potential regulators of R-loop dynamics in zygotes. These datasets were downloaded, reanalyzed, and compared with mass spectrometry data to identify candidate factors involved in regulating R-loop dynamics. To validate the functions of these candidate factors, we treated mouse zygotes with chemical inhibitors using *in vitro* fertilization. Immunofluorescence with an anti-R-loop antibody was then performed to quantify changes in R-loop metabolism.

**Results:**

We identified DEAD-box-5 (DDX5) and histone deacetylase-2 (HDAC2) as candidates that potentially regulate R-loop metabolism in oocytes, zygotes and two-cell embryos based on change of their gene translation. Our analysis revealed that the DDX5 inhibition of activity led to decreased R-loop accumulation in pronuclei, indicating its involvement in regulating R-loop dynamics. However, the inhibition of histone deacetylase-2 activity did not significantly affect R-loop levels in pronuclei.

**Conclusion:**

These findings suggest that dynamic changes in R-loops during mouse zygote development are likely regulated by RNA helicases, particularly DDX5, in conjunction with transcriptional processes. Our study provides compelling evidence for the involvement of these factors in regulating R-loop dynamics during early embryonic development.

## INTRODUCTION

Remodeling of the chromatin architecture has been identified as a crucial driving force for zygotic gene expression in various species [1,2]. A recent study highlighted the role of nucleosome-free regions and histone acetylation, particularly H3K9ac and H3K27ac, in the activation of genes involved in zygotic gene activation (ZGA), independent of DNA replication or transcriptional elongation [3]. These nucleosome-free regions are believed to be established through the binding of pioneer transcription factors that are necessary for ZGA. Additionally, the incorporation of RNA polymerase II (RNAPII) into the zygotic genome is associated with ZGA in mice [4,5]. Liu et al [5] demonstrated that RNAPII loading occurs in both activated and silenced genes during major ZGA and displays weak elongation activity during minor ZGA.

RNAPII occupancy is observed in both paused and productive elongation states during gene transcription [6–8]. Generally, RNAPII pausing inhibits transcription initiation and affects R-loop formation [9–12]. R-loops are DNA:RNA hybrids that are naturally synthesized *in vivo* and localized to the genome in a sequence-specific manner. R-loops play important regulatory roles in various cellular processes, including DNA replication, transcription, immunoglobulin G (IgG) class switch recombination, and DNA [13–17]. Although R-loops are potentially harmful structures that can lead to genomic instability and DNA damage, they are unavoidably formed during many cellular processes [18]. Their presence and proper regulation are critical for maintaining cellular homeostasis and preventing DNA damage [19,20]. Recent studies have identified R-loop-interacting proteins involved in replication, transcriptional regulation, alternative splicing, and DNA repair using mass spectrometry combined with immunoprecipitation (IP) with R-loop-specific S9.6 antibodies [21]. These proteins include RNA helicases, RNA processing factors, and chromatin remodelers [21,22]. Among these, a subset of RNA helicases belonging to the DEAH/RHA (DHX) and DEAD-box (DDX) families, particularly DDX5 and DHX9, have been identified as critical regulators of R-loop biogenesis and resolution [23–27]. Although the term “RNA helicases” implies unwinding RNA species, only a subset of them exhibits processive helicase activity [28–30]. Certain RNA helicases, including DDX1, DDX5, DDX17, DDX20, DDX21, and DHX9, have been implicated as coactivators or corepressors in transcriptional regulation [31]. In the case of DDX5, arginine methylation of its RGG/RG domain by protein arginine methyltransferase 5 (PRMT5) regulates R-loop levels, particularly at transcription termination sites [23,32]. The RGG/RG domain is required for interaction with RNAPII subunit A (POLR2A) and 5′-3′ exoribonuclease 2 (XRN2), facilitating the release of RNAPII and preventing R-loop formation [23,33–35].

Studies have demonstrated that the function of DDX5 in regulating R-loop dynamics is context-dependent [23,32,33, 36–38]. DDX5 promotes R-loop resolution under normoxia but promotes R-loop biogenesis under hypoxia [38]. Therefore, the precise role of DDX5 requires further investigation in different cell types, including preimplantation embryos. Although many studies have attempted to understand the biogenesis and resolution of R-loops and have demonstrated their diverse molecular functions *in vivo* and *in vitro*, very few studies have focused on the role of R-loops in early preimplantation embryos, particularly their involvement in the regulation of zygotic gene expression. In this study, we examined candidate factors that interact with R-loops using chemical inhibitors and found that RNA helicase DDX5 plays a critical role in R-loop metabolism in mouse zygotes.

## MATERIALS AND METHODS

### Metaphase II oocyte collection and *in vitro* fertilization

Female ICR mice aged 6 to 8 weeks and male ICR mice aged 8 to 10 weeks were obtained from Orient Bio Co., Ltd. (Seoul, Korea). All procedures involving mice were conducted in accordance with guidelines approved by the Institutional Animal Care and Use Committee of Konkuk University (IACUC approval number: KU22181). The mice were housed in a controlled environment at 22°C±1°C with a 12-h light-dark cycle.

To induce superovulation in female mice, intraperitoneal injections of pregnant mare serum gonadotropin (5 IU) were administered, followed by human chorionic gonadotropin (hCG; 5 IU) 48 h later. Fifteen hours after hCG injection, cumulus-oocyte complexes (COC) were collected from the oviduct and transferred to a modified human tubal fluid (mHTF) medium supplemented with 0.625 mM glutathione (GSH; Sigma-Aldrich, St. Louis, MO, USA). Spermatozoa were collected from the caudal epididymis of male mice and incubated in mHTF medium containing 0.4 mM methyl-β-cyclodextrin (MBCD)-polyvinyl alcohol (PVA) for at least an hour to induce sperm capacitation. Capacitated sperm was then added to the GSH-mHTF medium containing COCs. Two hours after insemination, the fertilized embryos were washed multiple times and cultured in EmbryoMax KSOM Mouse Embryo media (Merck Millipore, Burlington, MA, USA) at 37°C under 5% CO_2_.

### Treatment of inhibitors and immunofluorescence

Each inhibitor was added and incubated for 3 days, and dimethyl sulfoxide (DMSO; Sigma-Aldrich, USA) was used as a vehicle for inhibitors in the control group. Embryos were transferred to the KSOM medium containing either DMSO or a specific inhibitor at 4 h post-insemination (hpi) and cultured for 8 h (12 hpi). The following inhibitors were used in the present study: 5 or 100 nM trichostatin A (TSA; Sigma-Aldrich, USA) for inhibition of histone deacetylase 2 (HDAC2), 20 nM supinoxin (RX-5902; MedChemExpress, Monmouth Junction, NJ, USA) for DDX5 inhibition, and 100 μM 5,6-dichlorobenzimidazole 1-β-D-ribofuranoside (DRB; Sigma-Aldrich, USA) and 1 μM triptolide (TRP; Sigma-Aldrich, USA) for inhibition of transcription. After inhibitor treatment, the embryos were fixed with 4% paraformaldehyde in a solution containing 0.01% PVA in phosphate-buffered saline (PBS) for 15 min. They were then washed thrice with a washing buffer (0.05% Tween 20-0.01% PVA-PBS) for 10 min each. The embryos were permeabilized with 0.5% Triton X-100-PVA in PBS for 20 min, followed by three washes. The embryos were treated with 4 N HCl for 15 min to denature the DNA and then neutralized with 100 mM Tris-HCl (pH 8.5) for 20 min. Subsequently, the embryos were blocked by incubation with 5% bovine serum albumin (BSA)-PVA/PBS for 2 h. Primary antibodies, including anti-S9.6 antibodies (1:200, MABE1095; Sigma-Aldrich, USA) and anti-phospho-histone H2A.X (1:300, 2577; Cell Signaling Technology, Danvers, MA, USA), were diluted in 1% BSA-PVA/PBS and incubated with the embryos at 4°C overnight. After three washes, the embryos were incubated with secondary antibodies, including donkey anti-mouse IgG Alexa Fluor 488 conjugate (1:400; Life Technologies, Carlsbad, CA, USA) and donkey anti-rabbit IgG Alexa Fluor 546 conjugate (1:400; Life Technologies, USA), for 1 h in the dark. DNA was counterstained with 2 μM To-PRO3 Iodine (Thermo Fisher Scientific, Waltham, MA, USA) for 20 min. The stained embryos were washed several times and mounted on slides using a small drop of VECTASHIELD Antifade Mounting Medium (Vector Laboratories, Burlingame, CA, USA).

### Screening of candidate factors regulating zygotic R-loop metabolism

A publicly available bulk RNA-seq and low-input ribosome profiling (called LiRibo-seq) dataset [39] was used to examine the patterns of protein synthesis in mouse oocyte, 1-cell and 2-cell embryos. The dataset (GSE169632) was downloaded and reanalyzed, and the fragments per kilobase of transcripts per million mapped reads (FPKM) values of actively translated mRNAs were determined using DESeq2 tools. RNAPII ChIP-seq dataset (GSE135457) analyzed in oocyte, 1-cell and 2-cell embryos were used to identify the actively transcribed genes [5]. From the intersection of two independent IP-mass spectrometry (IP-MS) studies [21,22], a set of 205 proteins known to interact with R-loops was selected for further analysis based on their synthesis levels in the LiRibo-seq dataset.

### Image analysis

All images were captured using a laser confocal microscope (LSM 8000; Zeiss, Oberkochen, Germany). For 1-cell stage embryos, the middle optical section of Z-stack images containing the maternal and paternal pronuclei was selected for fluorescence intensity quantification. The fluorescence intensity of the S9.6 signal within the pronuclei was measured using the ImageJ software (National Institute of Health, Bethesda, MD, USA). Quantification was performed using the following approach: the total intensity of the pronucleus was calculated by multiplying the mean intensity of the pronucleus region by the area of the pronucleus and then subtracting the product of the mean intensity of the cytoplasmic region by the area of the pronucleus.

### Statistical analysis

Statistical analyses were performed using Prism software (GraphPad Inc., Boston, MA, USA). A two-tailed unpaired t-test with Welch’s correction was used to compare the two groups. One-way analysis of variance followed by Tukey’s test was performed for comparisons among more than three groups. The results are presented as mean±standard error of the mean or mean±standard deviation. Statistical significance is indicated as follows: * p<0.05, ** p<0.01, *** p<0.001, NS, not significant.

## RESULTS

### Identification of candidate factors that regulate zygotic R-loop metabolism

**Figure 1:**
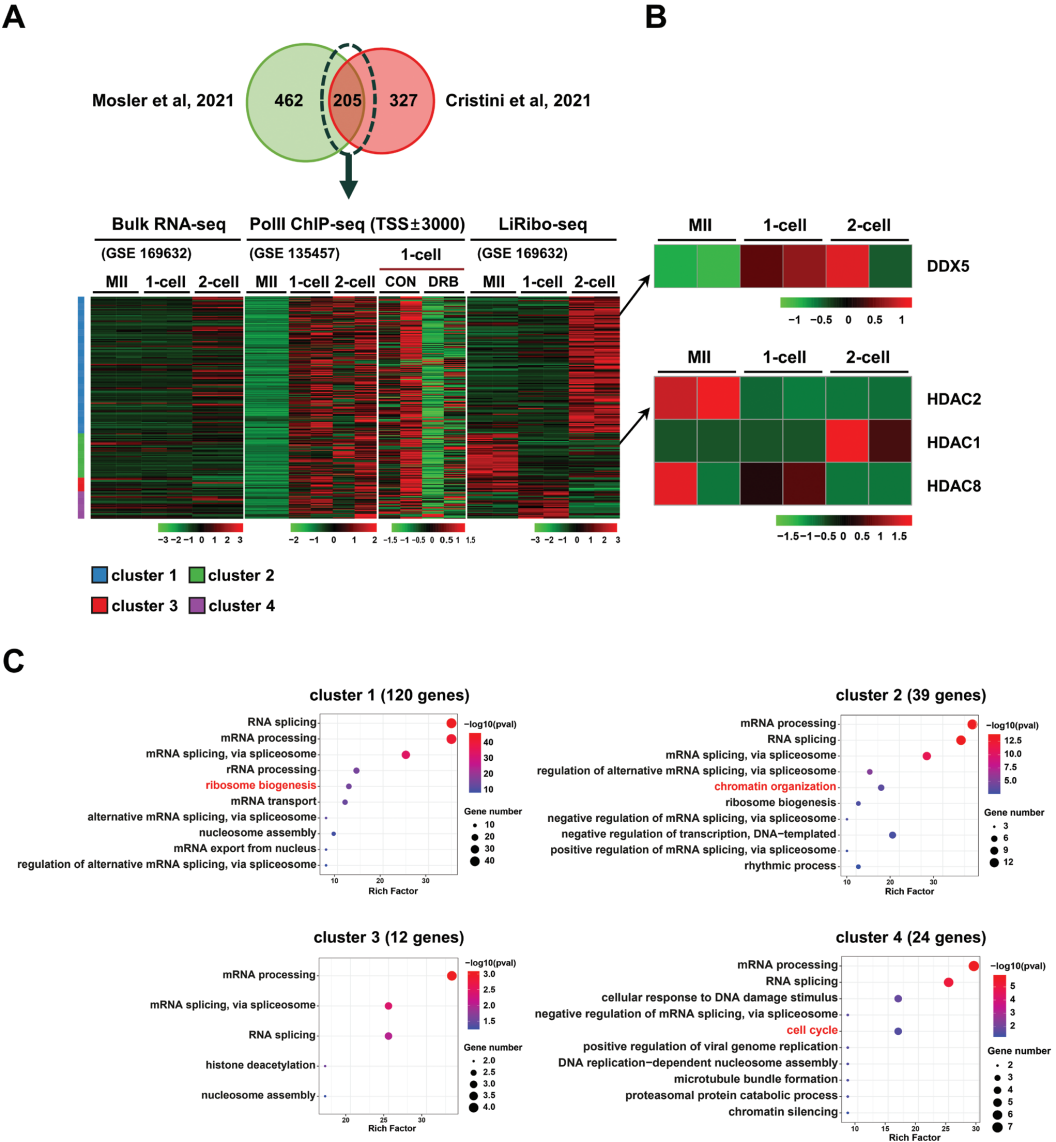
Identification of candidate factors that potentially regulate R-loop metabolism during zygotic genome activation. (A) Two hundreds five proteins were selected from two independent proteomics studies that identified R-loop-interacting proteins [21,22]. Publicly available bulk RNA-seq (GSE169632), RNAPII ChIP-seq (GSE135457), and low-input ribosome profiling (LiRibo-seq, GSE169632) datasets were analyzed. Heatmaps showing abundance of RNAs, enrichment of RNAPII at TSS ±3,000 bps, and differentially translating genes from zygote to 2-cell stage embryos. (B) Heatmaps showing translating RNA levels of DEAD-box-5 (DDX5) and histone deacetylase-2 (HDAC2). (C) Analysis of gene ontology (GO) of the differentially translating genes in cluster 1 (120 genes), cluster 2 (39 genes), cluster 3 (12 genes), and cluster 4 (24 genes).

To identify potential regulators of R-loop homeostasis in oocyte and early embryos, we analyzed publicly available LiRibo-seq (GSE169632) [39] and RNAPII ChIP-seq datasets (GSE135457) generated from mouse oocyte, 1-cell and 2-cell embryos [5]. From these datasets, 205 proteins that were previously identified as R-loop-interacting proteins [21,22] were selected (Figure 1A; Table 1), and their dynamic changes of gene translation from oocytes to 2-cell stage embryos were investigated. As depicted in Figure 1A, the levels of transcripts undergoing translation differed significantly from those detected in bulk RNA-seq, indicating a specific regulation of translation during early development. Hierarchical clustering analysis of the 205 proteins revealed four distinct clusters: cluster 1 (C1, 120 genes) displayed active translation at the 2-cell stage, C2 (39 genes) exhibited active translation at the oocyte stage, C3 (12 genes) showed active translation at both the oocyte and 1-cell stages, and C4 (24 genes) displayed active translation at the 1-cell stage. Additionally, enrichment of RNAPII at the transcription start sites (TSSs) was observed for most of the 205 genes, yet the RNAPII enrichment was greatly diminished upon DRB treatment (Figure 1A). Based on the translation profiles of these genes, we selected two candidate proteins known for their roles in RNA unwinding, DNA damage repair, and histone modification from the gene clusters (Figure 1B) and employed chemical inhibitors to investigate their functions in the regulation of R-loop metabolism during the ZGA period. The inhibitors used in this study were TSA, which inhibits HDAC2 (a gene from cluster C2), and supinoxin, which inhibits DDX5 (a gene from cluster C4). Interestingly, other class I HDAC genes displayed a distinct pattern of translation in the stages (Figure 1B). Gene ontology (GO) term analysis indicated that the genes within these four clusters were mainly associated with RNA processing and splicing, suggesting their involvement in RNA biology (Figure 1C).

### Zygotic R-loop dynamics links to gene transcription

**Figure 2:**
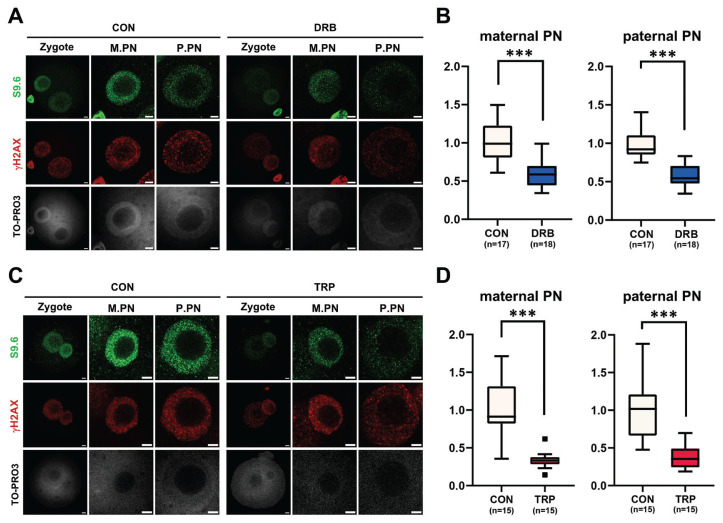
Dynamics of R-loop metabolism in response to inhibition of gene transcription. (A) Representative immunofluorescent images using anti-S9.6 (green) and anti-gH2A.X (red) antibodies after treatment with 100 μM 5,6-dichlorobenzimidazole 1-β-D-ribofuranoside (DRB), and quantification of their intensities (B). TO-PRO3 was used for the staining of nuclear DNAs. (C) Representative immunofluorescent images using anti-S9.6 (green) and anti-gH2A.X (red) antibodies after treatment with 1 μM triptolide (TRP) and quantification of their intensities (D). TO-PRO3 was used for the staining of DNAs. Scale bars, 5 μm. M.PN, maternal pronucleus. P.PN, paternal pronucleus. CON, Control. Graphs in (B) and (D) represent the mean and standard error mean. *** p<0.001.

In our previous study, we established a correlation between zygotic R-loop dynamics and gene transcription. To validate the finding, additional experiments were performed using DRB (Figure 2A, 2B), a transcription elongation inhibitor, and TRP (Figure 2C, 2D), a transcription initiation inhibitor. After *in vitro* fertilization, 1-cell embryos were treated with either TRP or DRB. Consistently, the transcriptional inhibition with either inhibitor significantly reduced R-loop formation in both maternal (M) and paternal (P) pronuclei (PN) (Figure 2B, 2D).

### HDAC inhibition causes no change in R-loop homeostasis

**Figure 3:**
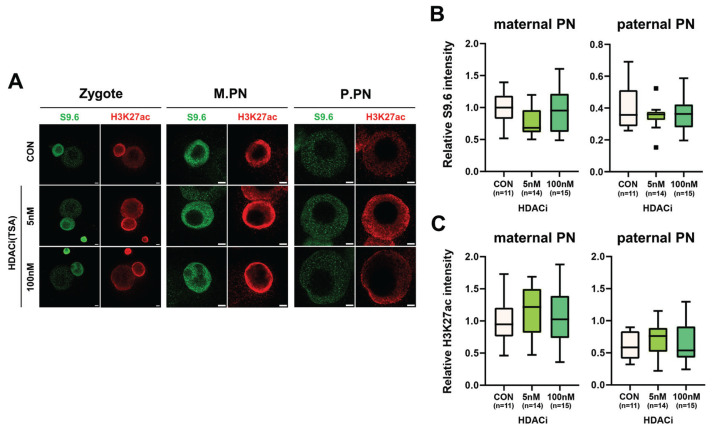
Effect of histone deacetylase-2 (HDAC2) inhibition on R-loop metabolism. (A) Representative immunofluorescence using anti-S9.6 (green) and anti-H3K27ac (red) antibodies after treatment with different concentrations (5 or 100 nM) of trichostatin A (TSA). (B, C) Quantification of signal intensities of immunofluorescence of S9.6 (B) and H3K27ac (C). M.PN, maternal pronucleus. P.PN, paternal pronucleus. Scale bars, 5 μm. CON, Control. Graphs represent the mean and standard error mean.

Given that the level of translated HDAC2 was high in and diminished in 1-cell and 2-cell embryos (Figure 1A), and HDAC2 function has been implicated in the ZGA [40], 1-cell embryos were treated with TSA to determine the role of HDAC2 in regulating R-loop formation during minor ZGA. The TSA treatment had little or no effect on the R-loop levels in both the maternal and paternal pronuclei of 1-cell embryos (Figure 3A, 3B). We also found that the levels of histone H3K27ac were slightly increased by both low and high concentrations of TSA (Figure 3A, 3C).

### Inhibition of RNA helicases impairs R-loop homeostasis

**Figure 4:**
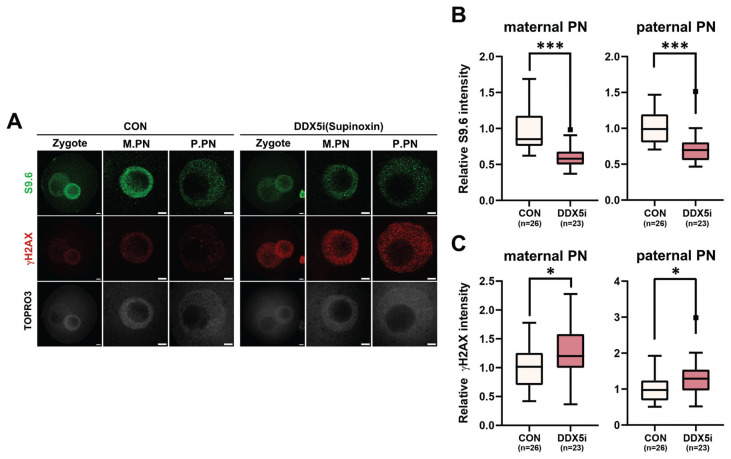
Effect of DEAD-box-5 (DDX5) inhibition on R-loop metabolism. (A) Representative immunofluorescence using anti-S9.6 (green) and anti-gH2A.X (red) antibodies after treatment with 20 nM supinoxin. (B, C) Quantification of signal intensities of immunofluorescence of S9.6 (B) and γH2AX (C). M.PN, maternal pronucleus. P.PN, paternal pronucleus. Scale bars, 5 μm. CON, control. Graphs represent the mean and standard error mean. * p<0.05. *** p<0.001.

Next, to investigate the function of RNA helicases in R-loop formation during minor ZGA of mouse zygotes, 1-cell stage embryos were treated with supinoxin (RX5902), an RNA helicase DDX5 inhibitor. The DDX5 inhibition [23] led to a notable reduction in the number of R-loop foci observed in 1-cell embryos (Figure 4A, 4B), whereas its inhibition elevated γH2AX level in both maternal and paternal pronuclei (Figure 4A, 4C). These results provide compelling evidence that RNA helicases, specifically DDX5, play critical roles in the regulation of R-loop metabolism in 1-cell embryos during minor ZGA.

## DISCUSSION

In this study, we identified candidate factors that may be involved in the production and resolution of R-loops and validated the function of selected factors using chemical inhibitors during the development of early preimplantation embryos. Two hundred and five candidate factors that potentially regulate R-loop formation were identified through DNA:RNA hybrid MS combined with IP studies using the S9.6 antibody and hybrid binding domain of RNase H1 (Figure 1A) [21,22]. In particular, the translation levels of these proteins exhibited different patterns as determined by low-input ribosome profiling analysis (Figure 1B). Proteins were divided into four clusters based on the translation status from MII oocytes to the 2-cell embryo stage; HDAC2 and DDX5 were selected from clusters 2 and 4, respectively. Chemical inhibitors targeting these candidates were used to investigate their effects on the R-loop biogenesis in minor ZGA.

A recent study showed that both HDAC1 and HDAC2 are required to regulate H3K27ac levels and ZGA in bovines [40]. Furthermore, deletion of both genes in growing oocytes leads to follicle development arrest at the secondary follicle stage [41]. To examine the effect of HDAC2 on R-loop formation in zygotes, R-loop levels were analyzed by immunofluorescence using the S9.6 antibody with and without treatment with TSA (an HDAC inhibitor). Although a slight increase in histone H3K27ac level was observed in the low TSA-treated group, this increase was not statistically significant. Notably, R-loop levels were not affected by the inhibition of HDAC2, regardless of the TSA concentration used. During ZGA, HDAC plays a crucial role in the regulation of H3K27ac, a marker of active promoters. The levels of H3K27ac dynamically change during this stage and increase in zygotes [42,43]. Our analysis suggests that aberrant H3K27ac levels resulting from HDAC inhibition do not affect R-loop formation in 1-cell embryos.

Timely and safe development of life during the preimplantation stages relies on the shift of transcripts from the oocyte to newly synthesized ones from the zygotic genome, a process known as maternal-to-zygotic transition (MZT) that occurs during ZGA [4,44,45]. Despite extensive research, the precise mechanism of MZT action remains unclear. The MZT is characterized by the transition of translational transcripts from maternal to zygotic origins [45]. The proper occurrence of minor and major ZGA is essential to ensure MZT. Pharmacological interference with transcription results in aberrant gene expression and developmental arrest [4]. Additionally, minor ZGA is closely associated with histone H3K4 methylation, as loss of H3K4 methylation impairs minor ZGA in the paternal pronucleus [46].

Following fertilization, a punctuated pattern of R-loop distribution throughout the genome has been observed in the zygotic pronuclei [47]. Although R-loop homeostasis is associated with both DNA replication and transcription, no study has elucidated the factors known to regulate R-loop metabolism in the minor ZGA of mouse zygotes.

Recent studies have provided evidence that DDX5 depletion in cells leads to the accumulation of R-loops near TSSs [33]. Furthermore, arginine methylation of the RGG/RG motif of DDX5 by PRMT5 and XRN resolved R-loops in U2OS cells [33]. Inhibition of DDX5 during the 1-cell stage resulted in a significant decrease in R-loop levels in both maternal and paternal pronuclei (Figure 4A, 4C), accompanied by an increase in γH2AX levels, a marker of DNA damage. Studies have demonstrated that DDX5 inhibition increases γH2AX levels, indicating its role in DNA damage repair [36,37]. Downregulation of DDX5 in embryos impedes normal pronucleus formation and development [48]. Therefore, defects in pronuclear development caused by DDX5 inhibition may lead to increased DNA damage and suppressed R-loop formation. This finding is consistent with those of our previous study, which showed that zygotes failed to promote R-loop formation during DNA damage repair.

The decreased S9.6 intensity observed in DDX5-inhibited zygotes can be explained by the reduced transcriptional activity of mature oocytes, which is maintained until ZGA occurs. Maternal RNA processing plays a crucial role during early development, particularly in the 1-cell stage embryo. A large proportion of proteins that interact with R-loops are involved in RNA splicing, processing, and ribosome biogenesis (Figure 1C). This suggests that DDX5 helicases unwind mRNA secondary structures and interact with R-loops for normal pronuclear development in the 1-cell stage embryo. Therefore, the decreased R-loop level observed in DDX5-inhibited zygotes may be attributed to reduced helicase activity, which hinders RNA strand displacement from the non-template DNA, thereby inhibiting R-loop formation.

Our analysis of the LiRibo-seq data revealed that GO terms enriched across all clusters were predominantly associated with RNA processing, highlighting the important role of RNA helicases and their relationship with R-loops in early embryonic development. As shown in (Figure 1C), RNA helicases, which play a role in unwinding RNA molecules, have been implicated in various RNA processing events, including transcription, RNA splicing, and RNA transport, by modulating RNA-RNA, RNA-DNA, and RNA-protein interactions. The DEAH/RHA (DHX) and DEAD-box (DDX) families of RNA helicases have been shown to regulate R-loop biogenesis and influence genome stability and DNA damage repair.

Notably, the function of DDX5 may vary depending on the cellular context. Although DDX5 has generally been implicated in R-loop resolution [23,32,33,36–38], recent studies have shown its involvement in R-loop formation under hypoxic conditions [38]. Therefore, it would be interesting to investigate how the function of DDX5 in zygotes differs from that in normal somatic cells. Additionally, the identification and functional validation of other factors that regulate DDX5 is crucial for a comprehensive understanding of its role in R-loop regulation.

## Figures and Tables

**Table 1:** List of candidate genes regulating R-loop metabolism

Genes (205)						
*Prpf40a*	*Raly*	*Ddx18*	*Ddx24*	*Gpatch4*	*Bms1*	*Pinx1*
*Gnl3*	*Ddx27*	*Luc7l2*	*Alyref*	*Hnrnpa3*	*Hnrnph2*	*Hnrnpa1l2*
*Ncoa5*	*Rbm14*	*Tra2a*	*Rrp12*	*Elavl1*	*Aatf*	*Mybbp1a*
*Bub3*	*Hnrnpf*	*Strbp*	*Hnrnpul2*	*Ebna1bp2*	*Srsf5*	*Tcof1*
*U2surp*	*Tra2b*	*Hist1h2bc*	*Hist1h2ab*	*Sart3*	*Sf3b2*	*Hist2h2ac*
*Rbm15*	*Gtpbp4*	*Rbbp7*	*Luc7l3*	*Wdr36*	*U2af1*	*Srsf4*
*Hnrnph1*	*Rrp1*	*Rbm22*	*Rbbp6*	*Ccdc86*	*Utp14a*	*Ptbp1*
*Ddb1*	*Srsf10*	*Coil*	*Ddx17*	*Tsr1*	*Uhrf1*	*Ddx50*
*Mta2*	*Hist1h2aj*	*Eftud2*	*Acin1*	*Hist1h2bm*	*Erh*	*Snw1*
*Rbm4b*	*Srsf6*	*Ncbp1*	*Srsf2*	*Rbm39*	*Nxf1*	*Heatr1*
*Sltm*	*Hist1h2ad*	*Snrpn*	*Dnttip2*	*Plrg1*	*Rrp1b*	*Incenp*
*Tpx2*	*Prpf19*	*Hist1h2bl*	*Hist1h2ah*	*Snrpb*	*Dkc1*	*Hnrnpc*
*Matr3*	*Skiv2l2*	*Sart1*	*Zc3h11a*	*Hnrnpk*	*Rbm25*	*Pes1*
*H2afj*	*Smarca4*	*Hist1h2ac*	*Ssrp1*	*Mfap1*	*Ahctf1*	*Safb*
*Ddx5*	*Fip1l1*	*Ncl*	*Snrpe*	*Hist1h2bd*	*Hist1h2bh*	*Kif23*
*Hnrnpd*	*Hnrnpr*	*Krr1*	*Hist3h2a*	*H2bfs*	*Hist1h4a*	*Chtop*
*Prpf4*	*Srsf11*	*Hist2h2aa3*	*Nvl*	*Prkdc*	*Ilf3*	*Dhx9*
*U2af2*	*Baz2a*	*Ngdn*	*Mki67*	*Nol11*	*Supt16h*	*Mmtag2*
*Thrap3*	*Wdr43*	*Dhx15*	*Grwd1*	*Psip1*	*H2afx*	*Hdac2*
*Nat10*	*Son*	*Hp1bp3*	*Srsf9*	*Hist2h2bf*	*Hist1h2ag*	*Mcm3*
*Srsf7*	*Prpf8*	*Ddx54*	*Pdcd11*	*Numa1*	*Ddx41*	*Rbbp4*
*Sfpq*	*Nkrf*	*Tardbp*	*Rbmx*	*Hnrnpul1*	*Kri1*	*Ddx39b*
*Ilf2*	*Hnrnpm*	*Ddx21*	*Cbx3*	*Snrnp200*	*Hnrnpu*	*Cdc5l*
*Baz1b*	*Srsf1*	*Rcc2*	*Hist1h2bn*	*Cbx5*	*Hnrnpl*	*Parp1*
*Nop56*	*Npm1*	*Nop58*	*Ddx39a*	*Znf326*	*Knop1*	*Top1*
*Hist2h3a*	*Xrn2*	*Adar*	*Hnrnpa0*	*Znf207*	*Srsf3*	
*Mphosph10*	*Fus*	*Pop1*	*Smarca5*	*Dek*	*Srrm2*	
*Hnrnpa2b1*	*Nop2*	*Noc3l*	*Nono*	*Cebpz*	*Hnrnph3*	
*Dnmt1*	*Hnrnpab*	*Hist1h2bk*	*Rbm8a*	*Taf15*	*Hnrnpa1*	
*Snrnp70*	*Top2a*	*Eif4a3*	*Zfr*	*Mcm5*	*Ctcf*	
